# Extracellular p53 fragment re-enters K-Ras mutated cells through the caveolin-1 dependent early endosomal system

**DOI:** 10.18632/oncotarget.1550

**Published:** 2013-12-02

**Authors:** Sun-Hye Lee, Tae-Gyun Woo, Su-Jin Lee, Jin-Sik Kim, Nam-Chul Ha, Bum-Joon Park

**Affiliations:** ^1^ Department of Molecular Biology, College of Natural Science, Pusan National University, Busan, Korea (Republic of); ^2^ Department of pharmaco-engineering, College of Pharmacy, Pusan National University, Busan, Korea (Republic of)

**Keywords:** p53 core domain, Oncogenic K-Ras, Caveolin-1, Drug delivery

## Abstract

K-Ras mutation is detected in over 30% of human malignancies. In particular, 90% of human pancreatic cancers are initiated by K-Ras mutation. Thus, selective elimination of K-Ras mutated cells would be a plausible strategy to prevent or cure the malignancies. In our previous reports, it has been revealed that oncogenic K-Ras promotes the exocytosis of p53 with Snail. In this study, we have followed the final destination of extracellular p53, which is secreted by the Snail complex. Here we provide evidences that p53, exported from K-Ras-mutated cells, is specifically re-endocytosed by oncogenic K-Ras-containing cancer cells. The p53 DNA-binding domain directly associates with caveolin-1 and enters K-Ras mutated cells through early endosome-mediated endocytosis. Using a serial deletion approach, we revealed that a fragment of human p53 extending from 93-143 amino acids (AA) is responsible for binding with caveolin-1 and for endocytosis. In contrast, p53-Snail binding occurs at the 143-193 aa region. Finally, through *in vivo* study, we confirmed that injected recombinant p53 could be up-taken by tumor tissues, constructed by oncogenic K-Ras transformed MEF cells. In contrast, the tumors formed by H-Ras mutated MEF cells did not accumulate the injected p53 protein. These results indicate that the p53 fragment might be useful as a specific delivery tool into K- Ras mutated cells as well as a diagnostic method.

## INTRODUCTION

K-Ras is a frequently mutated human proto-oncogene in many kinds of human cancers such as lung, colon, and pancreatic cancer [1-5]. In particular, pancreatic cancers exhibit extremely high mutation rate of K-Ras, over than 90% [1, 3]. Thus, specific inhibition of oncogenic mutated K-Ras is an ideal strategy to treatment of human cancers such as pancreatic cancer. However, K-Ras mutation-targeted drugs have not been proposed until now. Thus, chemotherapy of these cancers is dependent on conventional treatment such as DNA damaging agents, despite severe side effect. To reduce side effects and mortality of K-Ras mutated cancers, more specific delivery methods to cancer cells should be developed. In addition, selective delivery of specific materials (including peptides or fluorescence dyes) into K-Ras mutated cells would be a useful diagnostic tool to detect early cancers or cancer-prone cells, particularly in pancreatic or lung cancer.

In recent, we revealed the molecular mechanism of oncogenic K-Ras-mediated p53 suppression through Snail [6]. Oncogenic K-Ras eliminates intracellular p53 through exocytosis with Snail. Thus, autoantibody against Snail seems to increase in the serum of K-Ras mutated cancer patients [7]. However, we did not observe an increase of p53 autoantibody, consistent with other's reports that p53 autoantibody is not relevant to p53 mutation status or cancer progression [8, 9]. These results indicate that differentially from Snail, which is trapped by immune cells and presented as an antigen, p53 is digested by extracellular proteases [7] and endocytosed by neighboring cells. However, detail molecular mechanism about p53 re-endocytosis has not been apparently demonstrated until now. In this study, we monitor the final destination of extracellular p53 protein using recombinant p53 (RP53) to mimic the secreted p53 from K-Ras mutated cells and reveal that K-Ras mutated cells re-uptake the p53 through caveolin-1-mediated endocytosis. We also show the possibility of p53 peptide as K-Ras mutated cancer-diagnostic tools through *in vivo* study. Our results strongly suggest that p53 core domain can be used for diagnostic marker or drug delivery shuttle for K-Ras mutated cancer cells.

## RESULTS

### The p53 core domain is specifically endocytosed by oncogenic K-Ras-dependent manner

**Figure 1:**
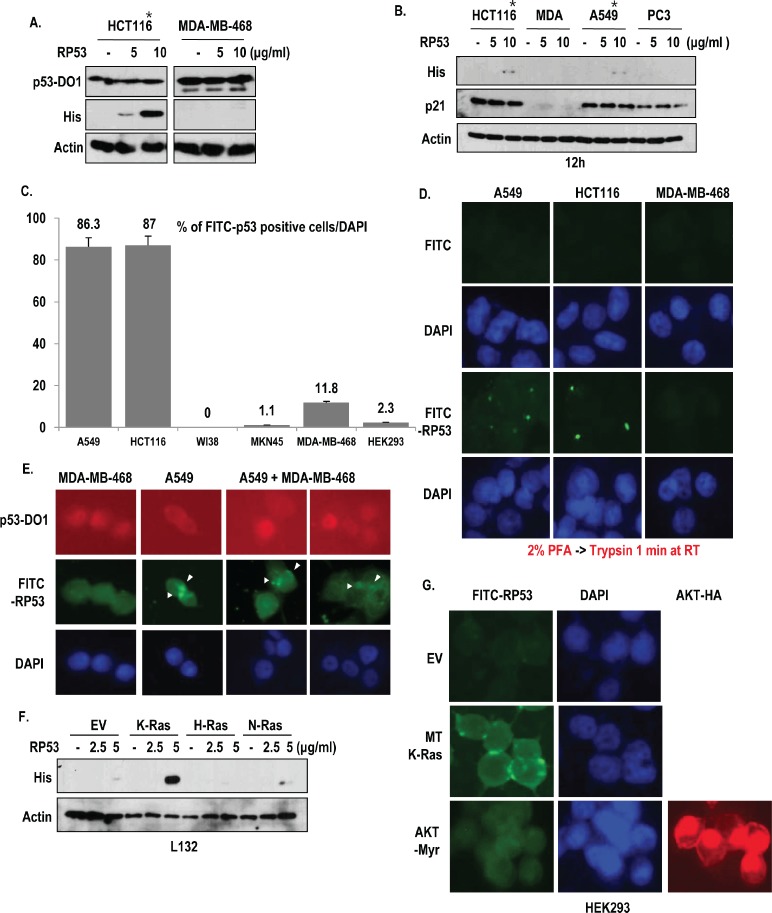
Specific uptake of RP53 by K-Ras mutant cell lines (A) Endocytosis of Recombinant p53 (RP53) occurred in mutant type K-Ras containing cancer cell lines (*) without alteration of endogenous p53 expression. RP53 was treated in HCT116 and MDA-MB-468 cells with indicating dosage. Cells were incubated with RP53 for 2 h. RP53 and endogenous p53 were detected using His antibody and DO-1, respectively. Actin was used as a loading control. (B) Selective endocytosis of RP53 in K-Ras mutated cells. Among 4 kinds of cells, K-Ras mutated cells (*) uptake RP53. Moreover, p21, target gene of p53, was not altered by RP53 treatment. (C) Cell counting of FITC-RP53 positive cells. To know how many cells could uptake RP53, we counted FITC positive cells from 200 cells. (D) RP53 incorporation was detected in cytoplasm of K-Ras mutated cells. 1 μg/ml of FITC and FITC-p53 was treated for 2 h in serum free condition. After fixation with 2% paraformaldehyde (PFA), cells were treated with Trypsin-EDTA for 1 min to eliminate non- specifically attached RP53. DAPI (4, 6-diamidino-2-phenylindole; blue) was used for nuclear staining. (E) In mixed cell lines, RP53 endocytosis specifically occurred in cells carrying mutant K- Ras. MDA-MB-468 (WT K-Ras/ MT p53) and A549 (MT K-Ras/ WT p53) cells were incubated with RP53 protein for 2 h. After fixation with MeOH for 20 min, cells were stained with p53-DO1 (Red), His (Green) and DAPI. (F) Endocytosis of RP53 occurred by K-Ras transfection in dose-dependent manner. Human embryonic cell L132 was transfected with K-, H- and N-Ras, and entered RP53 was detected by His antibody. (G) Endocytosis of RP53 was dependent on mutant K-Ras, but not activated AKT. After 2 h incubation of FITC-p53, cells transfected with EV, MT-K-Ras and AKT-Myr (Active form of AKT) were fixed with MeOH for 20 min and stained with HA (AKT; Red) and DAPI. Entered FITC-p53 was clearly observed in MT K-Ras overexpressed cells.

In the previous report, we have suggested that p53 is exocytosed from K-Ras mutated cells with Snail, which is captured by immune cell [7]. However, why released p53 is not recognized by immune system is remained to be elucidated. To address this, we generated recombinant p53 proteins (RP53; p53 DNA binding domain 93-292 amino acids (AA); His tag) and monitored the destination of RP53 after treatment into several types of human cancer cell lines. In spite of washing with PBS twice, extracellular treated RP53 was recovered from cell lysate only in K-Ras mutated cancer cell lines (*) but not in wild type K-Ras-cell lines including MDA-MB-468 (Breast cancer cell line; [12]), PC3 (Prostate cancer cell line; [13]) and MKN45 (gastric cancer cell line; [14]) (Figure 1A and B and Supplementary Figure 1A). In addition, we did not detect the RP53 up-take in non-cancer cell lines (WI38 and HEK293; Figure S1B), indicating that RP53 up-take is specific event of K-Ras mutated cells. However, treatment of RP53 did not alter the expression level of endogenous p53 (Figure 1A) or the expression of p21, a well-known target of p53 (Figure 1B). To trace the RP53 internalization directly, we labeled RP53 with FITC. Despite attachment of FITC alone in surface of MKN45 and MDA-MB-468 (Figure S1C), FITC-RP53 was not found in cytoplasm in these cell lines and also in non-cancer cell lines (WI-38 and HEK293). Instead, we could observe the intracellular localization of FITC-RP53 in A549 and HCT116 (Figure S1C). We calculated FITC-RP53 positive cells through cell counting and revealed that most of A549 and HCT116 cells contained FITC-RP53 (over than 85%; Figure 1C). In contrast, K-Ras WT-type cell lines did not show FITC signal (Figure 1C and Figure S1C). To avoid the artifact, resulted from adhesion of RP53 in cell surface, we eliminated the surface proteins by treatment of trypsin after fixation with 2% paraformaldehyde (PFA) and checked the localization of FITC-probes. Treatment of trypsin could eliminate non-specific binding of FITC on cell surface in MDA-MB-468 (Figure 1D). However, FITC-RP53 was still detected in A549 and HCT116 (Figure 1D). To confirm the K-Ras specific RP53 uptake, we co-cultivated two cell lines (A549; MT K-Ras and MDA-MB-468; WT K-Ras), and treated FITC-p53. After 2 h, we examined the location of FITC-RP53. Since MDA-MB-468 possesses highly stable p53 due to point mutation [15], whereas, A549 contains wilt type p53, which is extremely low stability, we could discern two cell lines by immunofluorence (IF) staining with N-terminal specific p53 Ab, DO-1 (Figure 1E). Consistent with our hypothesis and previous result (Figure 1B), FITC-RP53 was detected in p53-low cells (A549; Figure 1E). Our next question is whether mutant type K-Ras is enough for endocytosis of RP53. To address this, we transfected 3 kinds of Ras family into L132, human embryonic cell line, and monitored up-take of RP53. Among them, the transfection of oncogenic K-Ras, but not N- or H-Ras induced p53 up-take (Figure 1F). We also obtained the similar results from K-Ras transfected WI38 (Figure S1D). However, constitutively active AKT (AKT-Myr) did not elevate p53 up-take (Figure 1G). These results strongly suggest that oncogenic K-Ras is an essential factor for p53 endocytosis.

### Active endocytosis of p53 occurs in K-Ras dependent manner

**Figure 2:**
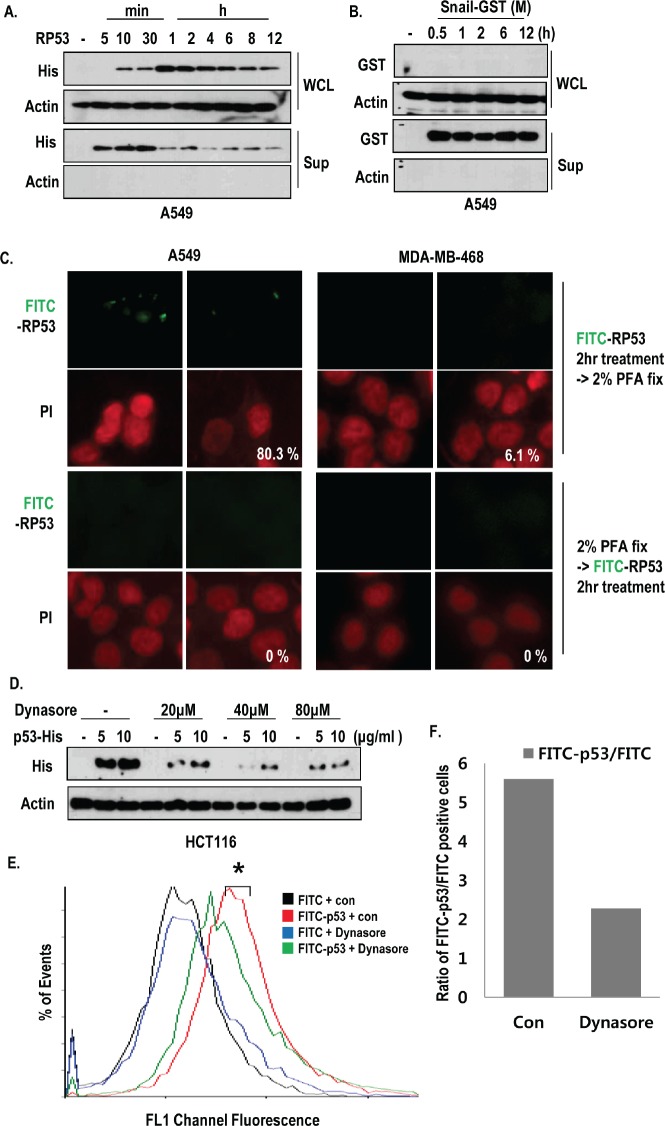
Active internalization of p53 core domain occurs by K-Ras dependent manner (A-B) K-Ras dependent endocytosis was a p53-specific event. RP53 and recombinant Snail (Snail middle region; GST tag; 91-121 amino acids (AA)) were treated in a time-dependent manner for up to 12h in the K-Ras harboring A549 cells. The endocytosis levels were measured by western blot analysis using the indicated antibodies. (C) Internalization of RP53 was appeared through active mechanism. Cells were divided into two groups; one group was incubated with FITC-p53 first and then fixed with 2% PFA, while the other group was exposed to PFA before FITC-p53 treatment. Both groups were stained with PI (propidium iodide; Red) for nuclear staining. (D) Dynamin inhibitor remarkably reduced endocytosis of RP53 in HCT116 cells. After incubation with Dynasore (from 20 to 80 μM) for 3 h, cells were treated with FITC or FITC-p53 for 1 h as an indicated dose. After washing with PBS, cells were harvested and subjected to SDS-PAGE. Western blot analysis was performed with indicated antibodies. (E) Decreased internalization of RP53 in A549 was measured by FACS analysis. (F) Internalized RP53 was quantified by graph. Numbers of Y-axis showed the value of FITC-p53 positive portion/FITC positive portion. After incubation with Dynasore (80 μM) for 3 h, cells were treated with FITC or FITC-p53 (2 μg/ml) for 1 h.

To know that oncogenic K-Ras may promote random endocytosis in regardless of protein species, we compared the endocytosis of Snail and RP53 in K-Ras mutated cells, time-dependently. The intracellular RP53 could be detected after 10 min, and it reached its maximum level after 1 h (Figure 2A). However, the recombinant Snail was not detected in A549 cells (Figure 2B), indicating that A549 cells selectively uptake RP53. In addition, we could observe the decrease of extra- and intracellular RP53 after 4 h (Figure 2A). To test whether RP53 uptake occurs through an active mechanism, we treated FITC-p53 into A549 and MDA-MB-468 cells before and after PFA-fixation. Pre-fixation could completely block the RP53 endocytosis (Figure 2C). In addition, treatment of RP53 at 4°C also blocked the internalization (Figure S1E). Since large GTPase, dynamin is essential for vesicle formation during overall endocytosis [16], we monitored the internalization of RP53 as dynamin dependent endocytic pathway using dynamin inhibitor; dynasore [17]. Treatment of dynasore was remarkably reduced the RP53 endocytosis as dose-dependent manner (Figure 2D). To analyze the quantification of endocytosed RP53, we measured FITC positive cells through FACS analysis and found that entered FITC-p53 was obviously decreased by dynamin inhibitor (Figure 2E and F). These results suggest that internalization of p53 is not non-specific event and occurs via active endocytosis pathway.

### The p53 core domain is up taken through caveolin-1 and early endosome

**Figure 3:**
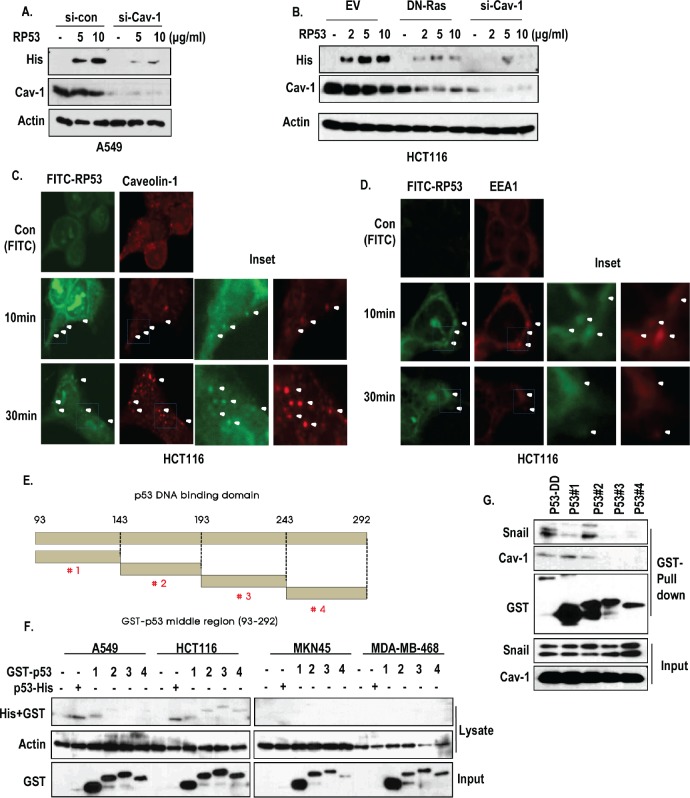
Caveolin-1-mediated endocytosis of p53 core domain (A) A549 cells, which were transfected with siRNA-targeting caveolin-1, were treated with RP53 for 2 h in a dose-dependent manner. Western blot analysis was performed with the indicated antibodies. Actin was used as a loading control. (B) Blocking of Ras activity or complete knock-down of caveolin-1 suppressed the endocytosis of RP53. HCT116 cells which were transfected with DN-Ras (Dominant Negative) and siRNA-targeting caveolin-1 were treated with RP53 for 2 h in a dose-dependent manner. Western blot analysis was performed with the indicated antibodies. Actin was used as a loading control. (C) Internalized RP53 was co-localized with caveolin-1 in time-dependent manner. HCT116 was treated with FITC or FITC-p53 following indicated time. After MeOH fixation, cells were stained with caveiolin-1 antibody (Red) and analyzed by confocal microscopy. Green (FITC) indicated endocytosed RP53. Inset indicated enlarged figure of boxed region. (D) EEA1, an early endosome marker, was also co-localized with treated RP53 in time dependent manner. Immunofluorence (IF) staining was performed with the protocol above, and the images were obtained using confocal microscopy. Inset indicated enlarged figures of boxed region. (E) Determination of key region for endocytosis through fragment mapping. The p53 DNA-binding domain (93-292 AA) was cleaved into 4 small fragments with 50 AA each, which were then tagged with GST. (F) Among the fragments, p53 #1 (93-142 AA) was specifically internalized into K-Ras mutated cell lines (A549 and HCT116). 4 cancer cell lines were incubated with either RP53 or with the 4 different fragments of p53. After 2 h, cells were washed twice with PBS and analyzed by western blot using the indicated antibodies. Actin was used as a loading control. (G) p53 #1 directly interacted to caveoin-1. Agarose bead- conjugated GST-p53 (DNA binding Domain; 93-292AA) and p53 fragments (p53 #1-4) were incubated with A549 cell lysates. GST-pull down was performed to monitor p53-associated proteins.

Since caveolin-1, which is increased by K-Ras [7, 20], is well-confirmed component of endocytosis pathway [18, 19], we focused on the role of caveolin-1 in RP53 endocytosis. As we expected, caveolin-1 knock down inhibited the endocytosis of RP53 (Figure 3A). In addition, blocking of Ras activity by DN-Ras could obviously reduce the expression of caveolin-1 and endocytosis of p53 (Figure 3B). To confirm the engagement of caveolin-1 in RP53 endocytosis, we performed IF staining with caveolin-1 and FITC-RP53 in HCT116. Because plasma-membrane-associated caveolin-1 can form the intracellular vesicles during endocytosis [18, 19], we monitored the caveolin-1-mediated vesicle formation, and found that internalized p53 was co-localized with caveolin-1 in cytoplasmic vesicles in a time-dependent manner (Figure 3C and Figure S2A). Consistent with our previous result (Figure 2A), internalized p53, detected at early time point, seemed to be diffused and degraded at late stage (4 h) (Figure S2B). We could also observe the interaction between RP53 and caveolin-1 through GST-pull down assay and immunoprecipitation (IP) analysis (Figure S3A and B). Since caveolin-1-mediated endosomes are targeted to the early endosome by association with EEA1 [19, 21], we examined the involvement of early endosomal system through IF staining with a maker of the early endosome, EEA1, and found that EEA1 was co-localized with FITC-RP53 in HCT116 cells (Figure 3D and Figure S3C). In addition, we observed the interaction between RP53 and EEA1 by IP analysis (Figure S3D). Interestingly, the interaction between caveolin-1 and EEA-1 was decreased by RP53 (Figure S3D), suggesting that the endocytosed RP53 is transferred from caveolin-1 to EEA-1-relaetd early endosome.

### Mapping of the p53 fragments shows that 93-143 AA is required for endocytosis

To know fine region of RP53 for endocytosis, we divided the RP53 into 4 fragments, consisted of 50 AA (Figure 3E). Among them, #1 fragment was taken up by A549 and HCT116 cells, but not by MKN45 and MDA-MB-468 cells (Figure 3F). To confirm this, we checked the endocytosis of fragment #1 in A549 and MDA-MB-468 and obtained that similar result that it could be taken up by A549 in a dose-dependent manner (Figure S3E). In contrast, fragment #4 did not show dose-dependent endocytosis in A549 or HCT116 cells (data not shown). In addition, caveolin-1 was strongly associated with fragment #1 in GST-pull down assay (Figure 3G). In contrast, Snail was associated with 143-193 region of p53 (Figure 3G). Finally, we confirmed the endocytosis of p53 fragment #1 into HCT116, but not in MDA-MB-468 cells through IF staining (Figure S3F).

### Endocytosis of p53 core domain occurs *in vivo* by oncogenic K-Ras

**Figure 4:**
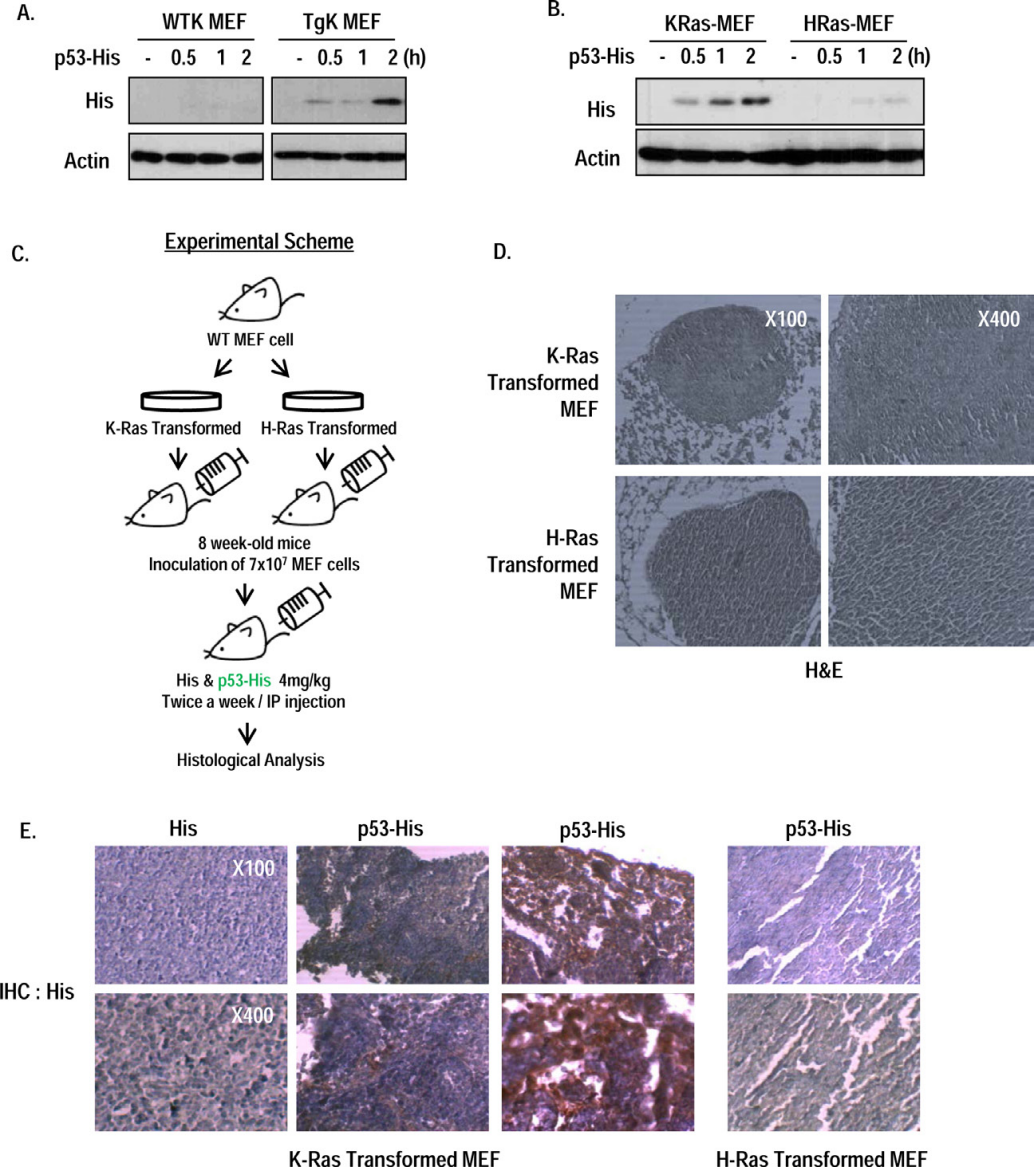
Oncogenic K-Ras dependent p53 endocytosis *in vivo* (A) RP53 was endocytosed into MEF cells obtained from K-Ras transgenic mouse (K-Ras LA2; TgK), but not from wild type mouse. After incubation with RP53 for indicated time, MEF cells were analyzed by western blot. Actin was used as a loading control. (B) Internalization of RP53 was found in K-Ras transformed MEF cells. MEF cells from wild type mouse were transformed with K- and H-Ras, and treated following indicated time. (C) Experimental scheme of in vivo study. Stabilized MEF cells expressing K- and H-Ras were inoculated into 8 week-old wild type C57BL/6 mice through intraperitoneal (i.p.) injection. After 3 weeks, mice were injected with 1mg/ml of His and His-p53 (RP53) 24 h before sacrifice. (D) Tumor mass was obtained from peritoneum of pancreas. Histological analysis was performed by standard hematoxylin and eosin (H&E) staning protocol. (E) Injected RP53 was endocytosed into tumors of K-Ras, but not H-Ras transformed MEF cell. Histological analysis was performed by immunohistochemistry(IHC) using His antibody to verify RP53 endocytosis.

To expand our study, we checked the RP53 endocytosis in MEF cells from wild type and K-Ras whole body transgenic mouse (K-Ras LA2 Tg; [10]); Up-take of RP53 was only shown in K-Ras Tg MEF (Figure 4A). To test that our result can be applied to in vivo diagnostic method, we designed the allograft assay using immortalized MEF cells by transformation of K- or H-Ras. After checking the non- toxicity and selective uptake of RP53 by K-Ras MEF cells (Figure 4B and Figure S4A and B), we inoculated immortalized MEF cell into 8 week-old-wild type C57BL/6 mouse via i.p. (intraperitoneal) injection to generate tumors of K- or H-Ras transformed MEF cells (Figure 4C). After 3 weeks, RP53 was administered twice a week by i.p. injection. After 24 h, we sacrificed mice and isolated tumor that was confirmed by hematoxylin and eosin (H&E) staining (Figure 4D). To monitor the endocytosed RP53 into tumor mass, we performed immunohistochemistry (IHC) analysis with His antibody. Notably, His-RP53 protein was detected in mice bearing K-Ras containing tumor cells, but not in H-Ras harboring cells (Figure 4E and Figure S 4C). However, His protein alone was not recovered in K-Ras transformed tumor cells (Figure 4E and Figure S 4C). Collectively, p53 endocytosis certainly occurs *in vivo* as well as *in vitro* by oncogenic K-Ras dependent manner, which reveals the possibility of developing the effective drug delivery system using this property of p53 endocytosis.

## DISCUSSION

In this study, we show that the p53 DNA binding domain (RP53) can be endocytosed by K-Ras mutated cell lines (Figure 1A and Figure S1A and B). Because K-Ras mutation occurs in over 30% of all human cancers (in particular, 90% of pancreatic cancer) [1-5], our peptide would be useful for delivery method for K-Ras mutated cancers. In fact, RP53 can be specifically entered into the K-Ras mutant cells (Figure 1D), and *in vivo* model (Figure 4E and Figure S4C). These results suggest that, if cell-impermeable chemicals that possess strong cytotoxicity were combined with RP53, K-Ras mutated cells could be selectively eliminated. Indeed, we are now searching the proper candidate for co-treated chemicals that cannot normally enter cells but possesses strong cytotoxicity. In addition, we revealed that p53 endocytosis is achieved through a different domain from Snail binding region (Figure 3G) and that Snail is not taken up by cells (Figure S3F). These results explain why Snail, which is co-exocytosed with p53, has a different destination [6]. When p53 and Snail are secreted from K-Ras mutant cells, a p53 fragment that would normally be processed by the ECM, may be taken up by neighboring cells. However, Snail is captured by immune cells. Although we have revealed that 93-143 AA is responsible for p53 endocytosis (Figure 3E and F), 50 AA is too large to use diagnostic tool or drug delivery shuttle. Thus, more intensive study and fine mapping should be performed for development of therapeutics or diagnostics tool. Taken together, the RP53 can be endocytosed by K-Ras mutant cells through caveolin-1/EEA-1- mediated early endosomal pathways and could be potentially used for a specific drug-delivery system through further improvement.

## MATERIALS AND METHODS

### Cell culture and reagents

Cell lines were obtained from American Type Culture Collection (ATCC, Manassas, VA) and maintained in 10% FBS-containing liquid media (RPMI-1640 or DMEM), supplied with 5% CO2 in a humidity chamber. MEF cells of wild type C57/B6 and K-Ras LA2 Tg mouse [10], were prepared for 13.5-day embryos through standard protocol [6].

### Recombinant proteins

The generation of recombinant proteins has been described in our previous report [6, 7]. The p53 DBD (RP53; DNA binding domain; 92-293 AA) protein was cleaved into 4 fragments. Each fragment was expressed in *Escherichia coli* as a GST-fusion protein, loaded onto GSH-agarose, and then eluted using a buffer containing 20 mM reduced glutathione after extensive washing. The eluted fractions were further purified using anion exchange chromatography (HitrapQ; GE Healthcare Biosciences, Piscataway, NJ). The p53 protein was labeled with NHS- Fluorescein, an amine-reactive derivative of fluorescein dye, according to the manufacturer's instructions (Thermo Scientific).

### Western blot analysis, GST pull-down assays and immunoprecipitation assays

Proteins were extracted from cells using a radioimmunoprecipitation assay (RIPA) buffer and subjected to SDS-PAGE, and then general western blotting (WB) analysis. Antibodies detecting His-tag (H-3; 1/1000), caveolin-1 (N-20; 1/1000), GST (B-14; 1/1000), and actin (I-19; 1/1000) were purchased from Santa Cruz Biotech (CA, USA.). Antibody against p21 (2946; 1/1000) was obtained from Cell Signaling Technology (Danvers, Mass., USA), and antibody against EEA1 (610457; 1/200) was from BD Biosciences (California, USA). Secondary antibodies used include HRP-linked goat anti-mouse, goat anti-rabbit, and mouse anti-goat (all 1/10000; Pierce, Thermo Fisher Scientific Inc., Rockford, IL, USA). To examine the physical interaction between proteins, immunoprecipitation assay and GST-pull down assay were performed [6, 7].

### Immunofluorescence staining

Cells, seeded on coverslips, were washed and fixed with 100% Me-OH or 2% PFA for 20 min. After fixation, cells were treated with trypsin for 1 min to eliminate proteins anchored to the outside of the cell membrane. After blocking with buffer (PBS/anti-Human Ab (1:500)), cells were incubated with primary antibodies (1:200; overnight at 4°C) and with rhodamine-conjugated secondary antibodies at 4°C for 2 h, sequentially. After staining the nuclei with either 4',6-diamidino-2-phenylindole (DAPI) or propidium iodide (PI), cells were mounted with mounting solution (H-5501; Vector Laboratories (Burlingame, CA, USA)) and analyzed by fluorescence microscopy (Zeiss) and confocal microscopy (Olympus FV10i Confocal).

### FACS analysis

Cells were seeded on 6 well plates and incubation with or without 80uM of dynasore 3 h prior to treatment of FITC and FITC-p53 (2 μg/ml). After 1 h, cells were washed with PBS and corrected by Trypsin-EDTA. Cells were fixed in 70% ethanol for 2 h at 4°C, and re-suspended in PBS containing propidium-iodide (50 μg/ml) followed by PBS washing. Analysis of 10,000 cells was performed on FACS (Beckman coulter).

### Transfection and si-RNA

For cell transfection, we used the jetPEI reagent according to the manufacturer's protocol [6, 7]. For an *in vitro* gene knockout, we generated si-RNA against caveolin-1 [11]. Using the jetPEI reagent, we transfected the si-RNA and checked the effect after 24 h.

### Measurement of cell viability

To examine the cell viability, cells were incubated with 0.5 mg/ml of MTT solution (Calbiochem, Darmstadt, Germany) for 4 h at 37°C. After removing excess solution and washing with PBS, the precipitated materials were dissolved in 200 ml DMSO and quantified by measuring absorbance at 540 nm.

### *In Vivo* tumorigenesis assay and Immunohistochemistry

All experimental procedures using laboratory animals were approved by the animal care committee of Pusan National University. Wild type C57BL/6 mice were purchased from Jackson laboratory (Maine, USA), maintained under temperature- and light-controlled conditions (20–23°C, 12 h/12 h light/dark cycle) and provided autoclaved food and water ad libitum. Each 8 week-old- mouse was inoculated intraperitoneally (i.p.) with 7X10^7^ cells of K- (K-Ras and SV40 small T) or H- Ras (H-Ras and SV40 Large T) transformed MEF. After 3 weeks for adaption, 50 mg/kg of His or His-p53 recombinant proteins were administrated 2 times a week by i.p. Injection. 24 h later, mice were dissected and histological tissue analysis was performed by basic procedure. In brief, sections were cut at 5 μm thickness and put onto an adhesive coated slide glass. For staining target protein, the slides were incubated with primary antibody against His (H-3; Santacruz), and secondary antibody (PI31430; Pierce) was treated for 4 h. DAB kit (SK-4100; Vector laboratories) was used to develop the slides.

## Supplemental Figure


