# Supplementary material for: Extracellular p53 fragment re-enters K-Ras mutated cells through the caveolin-1 dependent early endosomal system
Source: Oncotarget. 2013 Dec 2;4(12):2523–31. doi: 10.18632/oncotarget.1550 (PMC3926846; doi:10.18632/oncotarget.1550)

Supplementary Figure Legends

Supplementary Figure S1. K-Ras specific delivery of p53 core domain. (A-B) Among the tested cell lines, RP53 was endocytosed into only mutant K-Ras containing cells (*). Various kinds of cell lines, including a normal cell (WI38-VA13; Subclone 2RA), were incubated with RP53 for 2 h. Western blot analysis was then performed with the indicated antibodies. Actin was used as a loading control. (C) Entered RP53 was detected in K-Ras mutated cell lines (HCT116 and A549). FITC-p53 and FITC were treated in 6 different types of cell lines for 2 h, and washed with PBS before being harvested to avoid contamination. After fixation with MeOH, cells were stained with DAPI for nuclear staining and analyzed by microscopy. (D) RP53 uptake was induced by trans-fection of mutant form of K-Ras. Normal cell line (WI38- VA13; Subclone 2RA) was transfected with EV (empty vector) or MT-K-Ras and treated with RP53 for the indicated time. Endocytosed p53 was detected using the His antibody, and actin was used as a control. (E) Internalization of RP53 was impaired at the low temperature. HCT116 cells were incubated with RP53 at 4°C and 37°C for an hour. After MeOH fixation, entered RP53 was detected by His antibody.

Supplementary Figure S2. Caveolin-mediated endocytosis of p53 core domain. (A and B) Internalized RP53 was co-localized with caveolin-1 in time-dependent manner. HCT116 was treated with FITC or FITC-p53 following indicated time. After MeOH fixation, cells were stained with caveiolin-1 antibody (Red) and analyzed by confocal microscopy. Green indicated endocytosed RP53, and DAPI was used for nuclear staining.

Supplementary Figure S3. p53 endocytosis occurs via early endosome-caveolin-1 complex. (A) Caveolin-1 directly interacted to RP53. Agarose bead-conjugated GST and GST-p53 (DNA binding

Domain) were incubated with A549 lysates, and RP53-associated caveolin-1 was detected by western blot analysis after precipitation. (B) The binding between caveolin-1 and RP53 was confirmed through immuno-precipitation (IP) analysis. The lysates of A549 cells transfected with EV and caveolin-1 were incubated with RP53 for 2 h in a dose-dependent manner and then used for IP with anti-caveolin-1. Actin was used as a negative control. (C) Internalized RP53 was co-localized with EEA1 in time-dependent manner. Green indicated endocytosed RP53, and Red is EEA1. DAPI was used for nuclear staining. (D) RP53 showed the binding affinity with EEA1. After treatment with RP53, immunoprecipitation (IP) was performed with the EEA1 antibody in dose-dependent manner. Precipitated proteins were subjected to SDS-PAGE, and western blot analysis was performed using the indicated antibodies. Actin was used as a negative control. (E) p53#1 (9 3-142 AA) was internalized into A549 cells but not into MDA-MB-486 cells. After incubation with p53#1 fra gment for 2 h, cells were washed twice with PBS and analyzed by western blot using the indicated antibodies. Actin was used as a loading control. (F) Internalized p53#1 was shown in K-Ras mutated cells. After treatme nt with indicated proteins for 6 h, cells were washed using PBS. Following to fixing with MeOH, entered prot eins were detected by GST antibody. DAPI was used for nuclear staining.

Supplementary Figure S4. Oncogenic K-Ras dependent p53 endocytosis *in vivo.* (A) Endocytosed RP53 showed no effect on the cell viability. K- or H transformed MEF cells were treated with RP53 in dose-dependent manner. After 48 h, MTT assay was performed to measure the cell viability at 540nm. (B) RP53 was endocytosed into K-Ras transformed MEF cell in time-dependent manner, but not into H-Ras MEF cell. RP53 was treated following indicated time, and western blot analysis was performed with indicated antibodies. (C) Large amount of endocytosed RP53 was detected in tumor cells of K-Ras transformed MEF, but His alone was not taken up by K-Ras tumors. IHC analysis was performed with His antibody.

Figure S1

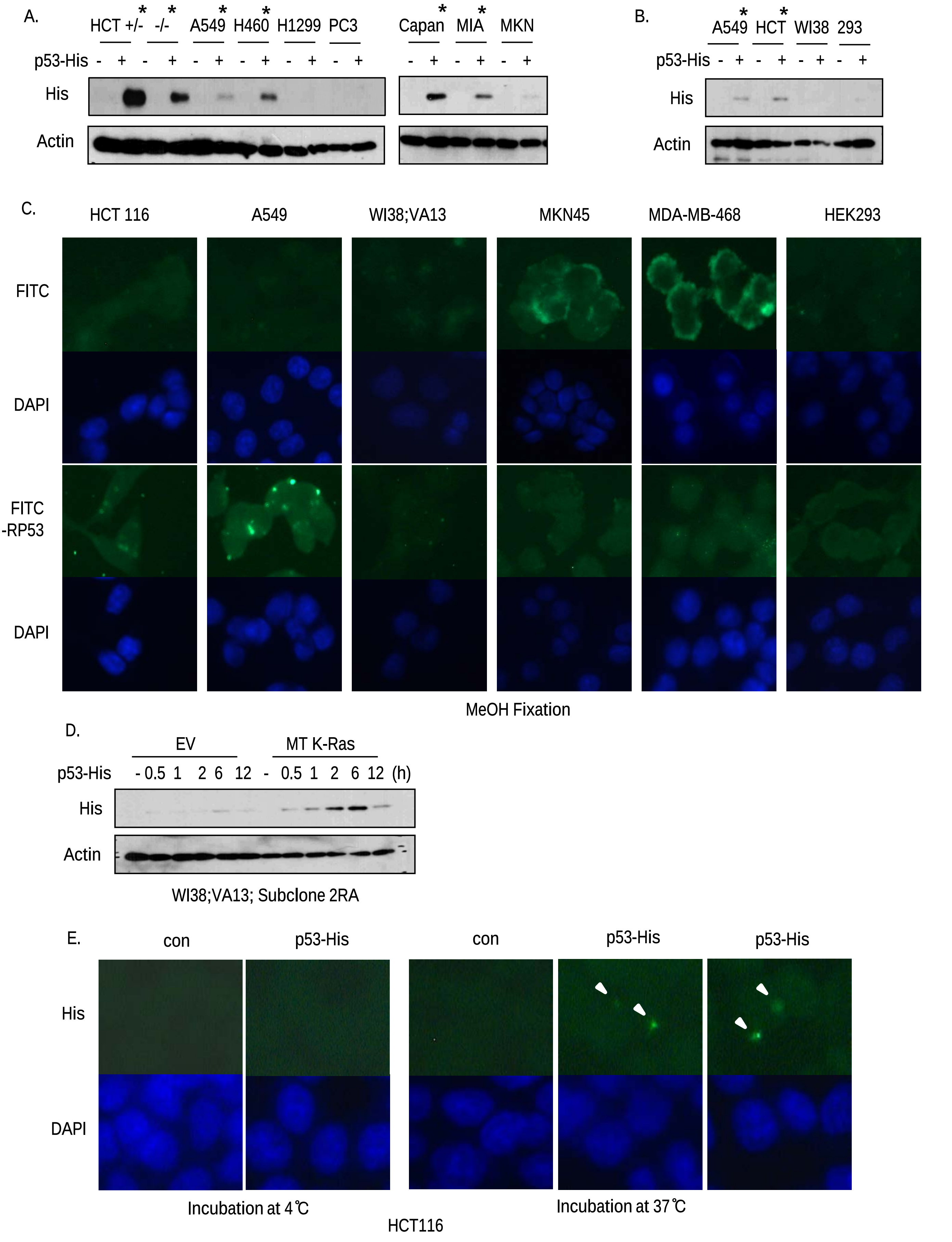

A.
HCT +/- * -/- * A549 * H460 * H1299 PC3
Capan * MIA * MKN
p53-His - + - + - + - + - + - +
His
Actin
B.
A549 * HCT * WI38 293
p53-His - + - + - + - +
His
Actin
C.
HCT 116
A549
WI38;VA13
MKN45
MDA-MB-468
HEK293
FITC
DAPI
FITC -RP53
DAPI
MeOH Fixation
D.
EV
MT K-Ras
p53-His - 0.5 1 2 6 12 - 0.5 1 2 6 12 (h)
His
Actin
WI38;VA13; Subclone 2RA
E.
con
p53-His
con
p53-His
p53-His
His
DAPI
Incubation at 4℃
Incubation at 37℃
HCT116

Figure S2

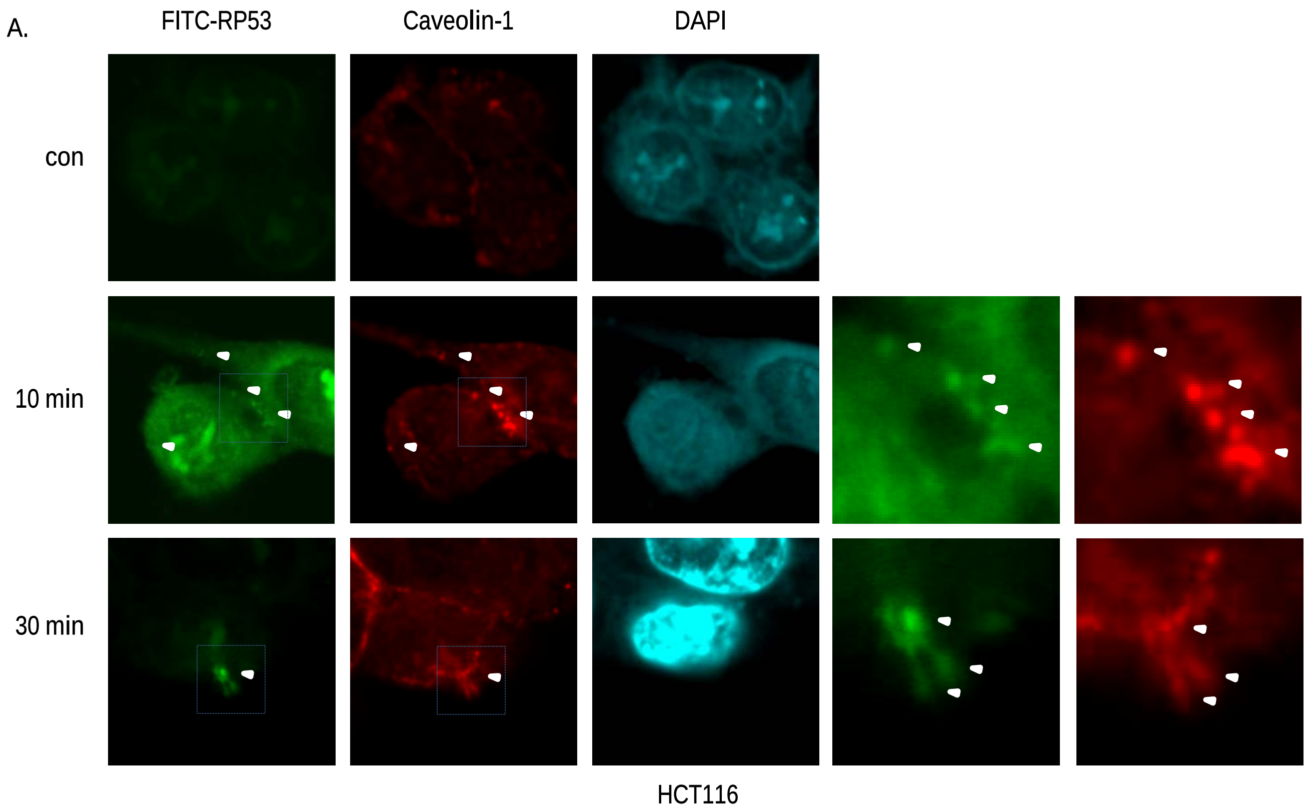
A.
FITC-RP53
Caveolin-1
DAPI
con
10 min
30 min
HCT116

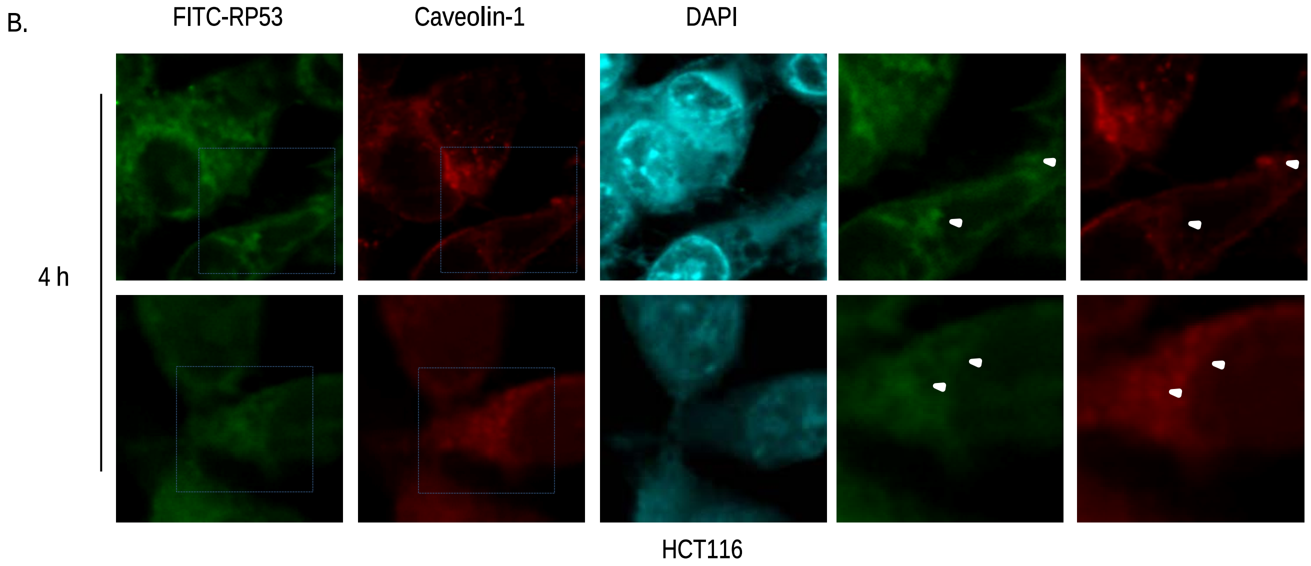
B.
FITC-RP53
Caveolin-1
DAPI
4 h
HCT116

Figure S3

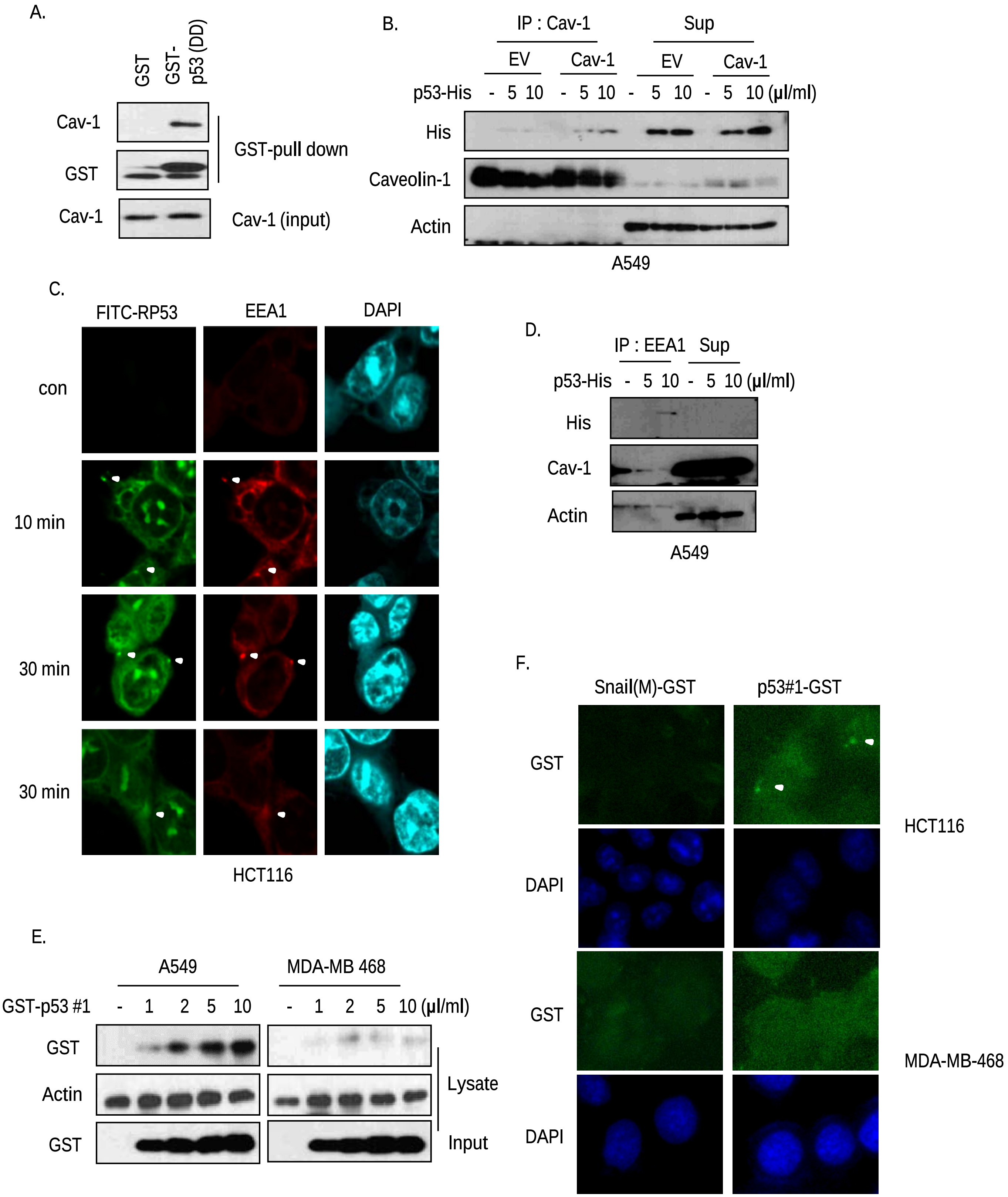
A.
GST
GST-p53 (DD)
Cav-1
GST
GST-pull down
Cav-1
Cav-1 (input)
B.
IP : Cav-1
Sup
EV
Cav-1
EV
Cav-1
p53-His - 5 10 - 5 10 - 5 10 - 5 10 (µl/ml)
His
Caveolin-1
Actin
A549
C.
FITC-RP53
EEA1
DAPI
con
10 min
30 min
30 min
HCT116
D.
IP : EEA1
Sup
p53-His - 5 10 - 5 10 (µl/ml)
His
Cav-1
Actin
A549
E.
A549
MDA-MB 468
GST-p53 #1 - 1 2 5 10 - 1 2 5 10 (µl/ml)
GST
Actin
Lysate
GST
Input
F.
Snail(M)-GST
p53#1-GST
GST
DAPI
HCT116
GST
DAPI
MDA-MB-468

Figure S4

A.

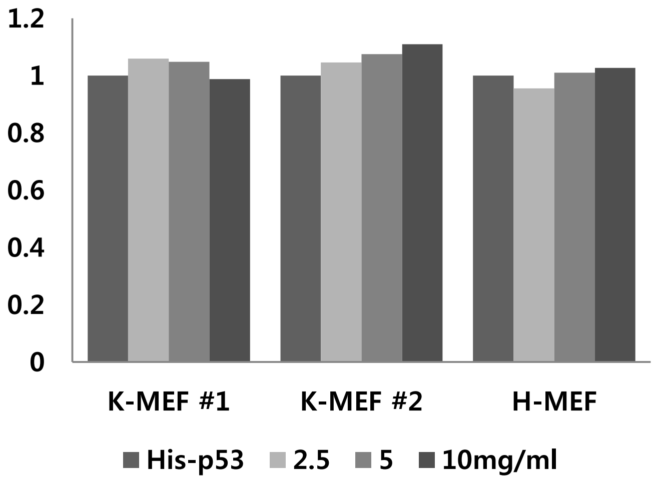

B.

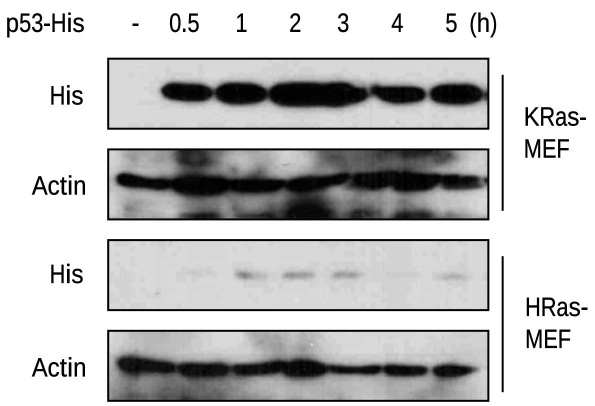

C.

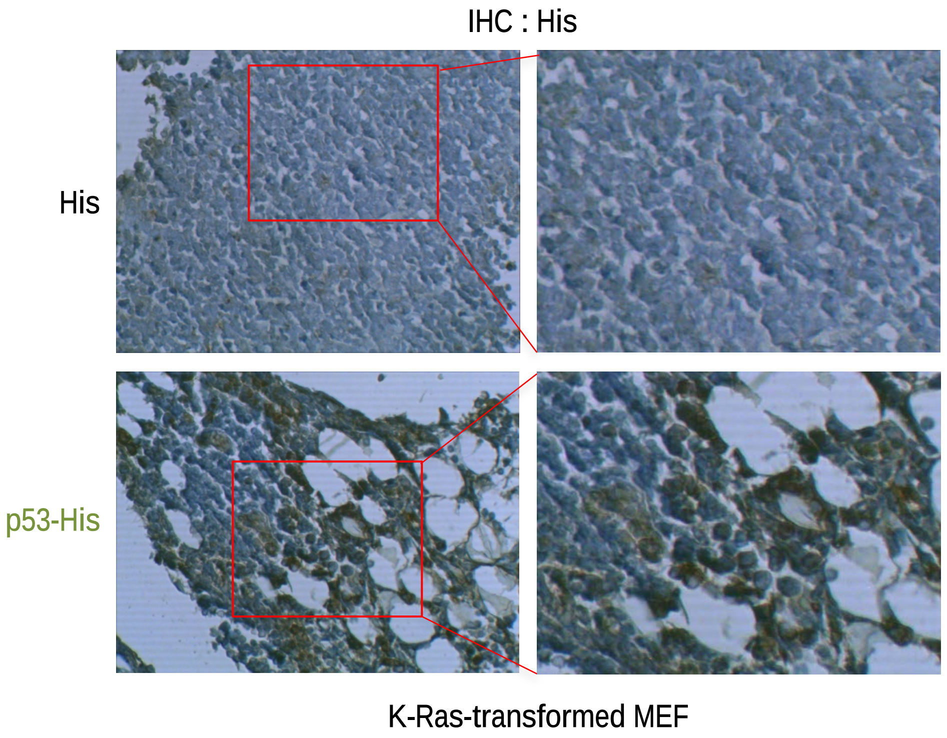

Supplement: Supplementary file 1 [file oncotarget-04-2523-s001.pdf]
